# Pair Bond-Induced Affiliation and Aggression in Male Prairie Voles Elicit Distinct Functional Connectivity in the Social Decision-Making Network

**DOI:** 10.3389/fnins.2021.748431

**Published:** 2021-10-15

**Authors:** Kyle R. Gossman, Benjamin Dykstra, Byron H. García, Arielle P. Swopes, Adam Kimbrough, Adam S. Smith

**Affiliations:** ^1^Department of Pharmacology and Toxicology, School of Pharmacy, University of Kansas, Lawrence, KS, United States; ^2^Department of Basic Medical Sciences, College of Veterinary Medicine, Purdue University, West Lafayette, IN, United States; ^3^Weldon School of Biomedical Engineering, Purdue University, West Lafayette, IN, United States

**Keywords:** functional connectivity, neural network, prairie vole (*Microtus ochrogaster*), social behavior, decision making, social decision making

## Abstract

Complex social behaviors are governed by a neural network theorized to be the social decision-making network (SDMN). However, this theoretical network is not tested on functional grounds. Here, we assess the organization of regions in the SDMN using c-Fos, to generate functional connectivity models during specific social interactions in a socially monogamous rodent, the prairie voles (*Microtus ochrogaster*). Male voles displayed robust selective affiliation toward a female partner, while exhibiting increased threatening, vigilant, and physically aggressive behaviors toward novel males and females. These social interactions increased c-Fos levels in eight of the thirteen brain regions of the SDMN. Each social encounter generated a distinct correlation pattern between individual brain regions. Thus, hierarchical clustering was used to characterize interrelated regions with similar c-Fos activity resulting in discrete network modules. Functional connectivity maps were constructed to emulate the network dynamics resulting from each social encounter. Our partner functional connectivity network presents similarities to the theoretical SDMN model, along with connections in the network that have been implicated in partner-directed affiliation. However, both stranger female and male networks exhibited distinct architecture from one another and the SDMN. Further, the stranger-evoked networks demonstrated connections associated with threat, physical aggression, and other aversive behaviors. Together, this indicates that distinct patterns of functional connectivity in the SDMN can be detected during select social encounters.

## Introduction

Close relationships represent significant aspects of our social world. In particular, marriage and intimate relationships are associated with extended life expectancy and better emotional and physical health compared to an absence of such relationships (Lillard and Waite, 1995; Kiecolt-Glaser and Newton, 2001; Robles and Kiecolt-Glaser, 2003; Rendall et al., 2011; Sbarra and Coan, 2018). However, these relationships can be challenged by a lack of commitment or unfaithfulness (Yamaguchi et al., 2015). It is critical that social partners display “commitment” through context-appropriate behaviors during social interactions, such as affiliation to a current partner and rejection (or aggression in some species) of other potential partners (Gonzaga et al., 2008).

It is theorized that these social decisions and others are processed in the brain through the Social decision-making network (SDMN). The SDMN hypothesis proposes that the expression of a social behavior is reflected by the overall activity of a network of brain structures rather than the activity of any single structure (Tremblay et al., 2017). The SDMN is comprised of thirteen brain regions (Table 1) that form two interconnected circuits including the social behavioral network and the mesolimbic reward system (O’Connell and Hofmann, 2011). The social behavioral network was first described in 1999 in mammals, and this network is comprised of the anterior hypothalamus (AH), bed nucleus of the stria terminalis (BNST), lateral septum (LS), medial amygdala (MeA), medial preoptic area (mPOA), periaqueductal gray (PAG), and ventromedial hypothalamus (VMH) (Newman, 1999). The social behavioral network has been suggested to regulate aggressive, sexual, and parental behaviors through sex steroid hormones. The second circuit in this network, the mesolimbic reward system, consists of the basolateral amygdala (BLA), BNST, caudate putamen (CP), hippocampus (HIP), LS, nucleus accumbens (NAcc), ventral pallidum (VP), and ventral tegmental area (VTA) (O’Connell and Hofmann, 2011). The mesolimbic reward system has been shown to play a role in generating motivation to seek reward, facilitate reinforcement, social choice and decision-making, and valence and salience to cues associated with these outcomes (Nestler and Carlezon, 2006; Thomas et al., 2008; Aragona and Wang, 2009). It is proposed that variance in the functional connectivity across the nodes of the SDMN during social encounters is associated with the processing of relevant social information and promoting context-appropriate responses. Studies have demonstrated functional connectivity in neural networks associated with social recognition (Tanimizu et al., 2017), aggression (Teles et al., 2015), positive social interactions (Rilling et al., 2018), and mating (Johnson et al., 2016; Kabelik et al., 2018). However, no study has determined how the SDMN regulates behavior necessary for attachment or commitment (O’Connell and Hofmann, 2012). Thus, it is our intention to examine the regional activity, interregional coactivity, and network connectivity in distinct social encounters that give rise to specific social behaviors associated with relationship commitment.

**TABLE 1:** List of all thirteen regions associated with the social decision-making network, along with the abbreviations of each regions.

**Abbreviation Region**	
AH	anterior hypothalamus
BLA	basolateral amygdala
BNST	bed nucleus of the stria terminalis
HIP	hippocampus
LS	lateral septum
MeA	medial amygdala
mPOA	medial preoptic area
NAcc	nucleus accumbens
PAG	periaqueductal gray
CP	caudate putamen
VMH	ventromedial hypothalamus
VP	ventral pallidum
VTA	ventral tegmental area

**FIGURE 1:**
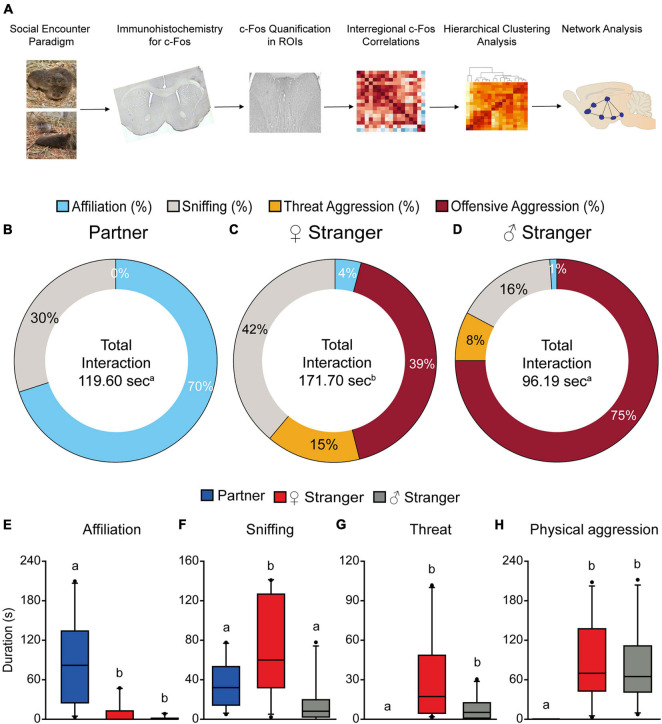
Schematic of experimental timeline **(A)**. Male prairie voles paired for 2 weeks induced selective affiliation toward a partner and aggression toward opposite and same sex stranger conspecifics. Male voles displayed significantly higher levels of affiliation compared to the other two social exposures **(B,E)**. Male voles spent significantly more time interacting with the opposite-sex stranger **(C)**, as well as higher levels of sniffing compared to the partner and same sex stranger **(F)**. Male voles spent the least amount of time interacting with the same sex stranger **(D)**. Male voles displayed higher levels of threat aggression **(G)** and offensive aggression **(H)** toward both the opposite-sex and same sex stranger. Groups labeled with unique alphabetical letters differed significantly by *post hoc* SNK test in which a significant main effect was detected in the ANOVA (*p* < 0.05). If groups share any letter, there was no significant difference between them. Thus, “a” was different from “b,” but “ab” was equal to both “a” and “b” (*n* = 10 per condition).

Although the SDMN is thought to be an evolutionarily conserved network across vertebrates, it remains unclear as to how this network regulates and implements responses of social behavior (O’Connell and Hofmann, 2012). Models that display these distinct social behaviors associated with relationship commitment are limited in animal research. The prairie vole (*Microtus ochrogaster*) is a socially monogamous rodent that forms long-term pair-bond between breeding pairs, which provides a unique model to characterize functionality in this network associated with social commitment (Aragona and Wang, 2004). The prairie vole has been used for over three decades to study the neurobiology of pair bonding, which has led to well-defined behavioral characterization of the commitment signals^1^ of the pair bond, including partner affiliation and stranger aggression (Smith et al., 2013). This creates an ethologically valid model to measure social behaviors that constitute the behavioral expression of a pair bond. Furthermore, a number of brain regions in the SDMN have been individually identified to be involved in the regulation of partner affiliation, stranger aggression, or both in prairie voles (Walum and Young, 2018). Regions including the AH (Gobrogge et al., 2017), LS (Liu et al., 2001), MeA (Kirkpatrick et al., 1994), mPOA (Curtis and Wang, 2003; Gobrogge et al., 2007), and VP (Lim and Young, 2004) have been associated with affiliative behaviors. Regions that have been associated with aggression toward unfamiliar conspecifics include AH (Gobrogge et al., 2007, 2009), BNST, NAcc (Aragona et al., 2006), VMH (Lischinsky and Lin, 2020), and VTA (Curtis and Wang, 2005). Among the other remaining regions, the CP (Burkett et al., 2011) and PAG (Walcott and Ryabinin, 2017; Simmons et al., 2021) have been shown to play a general role in social attachment, and lastly the HIP (Dedovic et al., 2009; Fanselow and Dong, 2010), along with the BLA (Numan and Young, 2016) has been associated with olfactory investigation, stress response, and other emotional behaviors in prairie voles. With the use of the prairie vole model, we first assessed the behaviors displayed by male prairie voles toward a familiar or novel social stimulus after 2 weeks of cohabitation with an opposite-sex partner (Figure 1A). Next, we assessed neuronal activation, using the immediate early gene c-Fos, during the various social exposures. The neuronal marker, c-Fos, is a well-established marker for regional activity and has been used in prairie voles to study such activity during aggression (Wang et al., 1997), affiliation (Gobrogge et al., 2007), mating (Curtis and Wang, 2003), and parental behaviors (Katz et al., 1999; Lang et al., 2019). Finally, we generated functional connectivity networks for each exposure and assessed the nature of each network in comparison to the theorized SDMN.

## Materials and Methods

### Animals

Subjects were captive-bred sexually naïve male prairie voles descended from populations captured in southern Illinois. Voles were weaned on postnatal day 21 and housed with a same-sex conspecific in cages (29.2 L × 19.1 W × 12.7 H cm) containing corn cob bedding and crinkle paper nesting material with food (LabDiet Rabbit Diet 5321) and water *ad libitum*. Colony rooms were maintained on a 12L:12D photoperiod (lights on at 0600 h) and at a temperature range of 21 ± 1°C. Subjects were 70–100 days of age at the start of the experiment. All procedures were conducted in accordance with the National Institutes of Health Guide and Use of Laboratory Animals and the Institutional Animal Care and Use Committee at the University of Kansas.

### Social Cohabitation and Resident Social Encounter Paradigm

The procedures used have been described elsewhere (Wang et al., 1997; Gobrogge et al., 2007). In brief, 40 total male subjects (10 per condition), aged 70–100 days were paired with sexually receptive females. Prior to establishing male-female pairs, females were estrogen primed with one daily subcutaneous injection of estradiol benzoate (EB) at 1 μg/100 μl sesame oil for 3 days consecutively, which successfully induced behavioral estrus. Pairs were housed together in plastic cages (29.2 L × 19.1 W × 12.7 H cm). Female partners were checked for vaginal plugs for the initial 4 days to ensure mating. On day 12, breeding pairs were transferred to larger cages (47.6 L × 26.0 W × 15.2 H cm) to establish residency. On the 15th day, male subjects were brought to the behavioral testing rooms and acclimated to their home cage for 5 min after their female partner was removed. Male subjects were randomly assigned to one of three social exposures for the resident social encounter test: (1) re-exposed to their familiar female, (2) exposure to an unfamiliar female, or (3) exposure to an unfamiliar male (both intruders age and size matched). Stimulus animals had a shaved patch on their back to distinguish them from the subjects. A fourth group of males was not exposed to any social stimulus to serve as the non-stimulated control. Behavioral interactions between the male subjects and the social stimulus were recorded for 10 min (between 0800–1100 h). Frequency and duration of affiliative and aggressive behaviors displayed by the resident was scored. This included olfactory investigation, side-by-side contact, huddling, allogrooming, attacks, bites, chases, defensive/offensive upright postures, offensive sniffs, threats, and retaliatory attacks. All behavior tests were manually scored by an experimenter blinded to experimental conditions using JWatcher (Blumstein and Daniel, 2007). The frequency and duration of affiliative and aggressive behaviors were analyzed by a one-way ANOVA (between subjects: condition). Pairwise comparisons between multiple groups were performed with Student-Newman-Keuls (SNK) *post hoc* analysis. Effects were considered significant at *p* < 0.05. After behavioral assessment, the social stimuli were immediately removed, and male subjects were housed alone for 2 h before being perfused (Winslow et al., 1993; Gobrogge et al., 2007). Female partners were sacrificed and dissected to determine pregnancy status. All females were determined to be 10–12 days pregnant based on previously validated methods in prairie voles (Aragona and Wang, 2009; Curtis, 2010).

### Tissue Preparation

Subjects were anesthetized with an i.p. injection of a ketamine / dexmedetomidine cocktail (75/1 mg/kg) then perfused through the ascending aorta with 15 ml of 0.9% saline, followed by 15 ml of 4% paraformaldehyde in 0.1 M phosphate-buffer (PB; pH 7.4). Brains were harvested, postfixed for 2 h in 4% paraformaldehyde at 4°C then stored at 4°C in 30% sucrose in PB for 3 additional days. Next, brains were cut into 30 μm coronal sections on a cryostat, and the slide-mounted tissue was stored in the −80°C freezer until processing for c-Fos immunohistochemistry.

### Immunohistochemistry

A 1:4 series through each region of interest from each brain was removed from the −80°C freezer and allowed to come to room temperature for 5 min. Sections were rinsed in 1× Phosphate Buffered Saline (PBS, 0.1 M, pH 7.4) for 15 min (3 × 5-min) then with 1% NaBH_4_ in 1× PBS at room temperature for 10 min. After rinsing in 1× PBS for 15 min, sections were blocked in 1× Powerblock (Universal Blocking Reagent 10×, Biogenex, Cat no. HK085-5K, Lot no. HK0850811) in 0.1% Triton X-100 for 30 min then incubated at room temperature for 30 min in rabbit anti-c-Fos polyclonal IgG (1:100, immunoStar, Cat no. 26209, Lot no. 1714001, RRID:AB_572267) in 0.05% TPBS (0.1 M PBS, 0.5% Triton X-100, pH 7.4) with 1% BSA then moved to 4°C for 48 h. Afterward, sections were rinsed in 0.05% TPBS for 5 min and then rinsed in 1× PBS for 10 min (2 × 5 min). Following, sections were incubated in 3% H_2_O_2_ in 1× PBS for 30 min then rinsed in 1× PBS for 15 min (3 × 5 min). The tissue was incubated at room temperature for 2 h in a goat anti-rabbit secondary antibody (1:150, Vector, Cat# BA-1000, Lot# ZF0809, RRID:AB_2313606). After, the tissue was rinsed in 1× PBS for 15 min (3 × 5 min) then incubated in VECTASTAIN ABC kit ((Peroxidase HRP), Cat# PK-4000, Lot# ZF1118) for 30 min. Next, the tissue was rinsed in 1× PBS for 15 min (3 × 5 min). Lastly, the tissue was stained with a DAB-Nickel Substrate Kit (Vector Lab, Cat# SK-4100, Lot# ZF0802) for 2.5 min, rinsed in 1× PBS for 15 min (3 × 5 min), and rinsed in ddH_2_O for 1 min. Slides were dried and cover-slipped.

### Imaging Quantification and Analysis

All tissue was imaged on a Leica DM6-B Microscope with a 10× objective. All regions were quantified bilaterally through ImageJ (Schindelin et al., 2012). Cell density (number of cells per mm^3^) of representative sections (3 brain sections) throughout each brain region was quantified by an experimenter blinded to experimental conditions and calculated. The cell counts for each condition was analyzed by a one-way ANOVA (between subjects: condition). Pairwise comparisons between multiple groups were performed with Student-Newman-Keuls (SNK) *post hoc* analysis. Effects were considered significant at *p* < 0.05.

### Neural Network Analysis Using Graph Theory

A graph theory approach was used to analyze the functional network connectivity between all brain regions that were examined. Graph theory is a branch of mathematics and computer science that explores the patterns of connectivity between multiple vertices within a system. Prior research has employed graph theory to analyze large datasets across a wide array of disciplines including organic chemistry, public transportation, finance, etc., (Garcia-Domenech et al., 2008; Derrible and Kennedy, 2009; Quintero et al., 2013; Qiang and Fan, 2016). In addition, several studies have demonstrated functional connectivity associated with social recognition (Tanimizu et al., 2017), aggression (Teles et al., 2015), mating (Johnson et al., 2016; Kabelik et al., 2018), and positive social interactions (Rilling et al., 2018; López-Gutiérrez et al., 2021). We have used a graph theory approach to generate network models of functional connectivity between multiple brain regions. This approach allowed for an investigation of the most important nodes within the social decision-making network.

In most network models, nodes are grouped together into clusters based on shared characteristics. For example, a network model that reflects airport flight paths may group airports together into clusters based off of region. In this experiment, we employed a hierarchical clustering approach to unbiasedly cluster brain regions together based on shared patterns of coactivity. Interregional c-Fos correlations were used to calculate the Euclidean distances between each pair of brain regions for each behavioral condition. Distance matrixes were then arranged by both row and column according to their shared patterns of coactivity. Dendrograms were then generated to reflect the Euclidean distance between each brain region. The dendrograms were cut at half the height of each tree to cluster the brain regions into groups of nodes called modules. The dendrogram threshold of 0.5 was chosen because it has been used in prior c-Fos network modeling experiments (Kimbrough et al., 2018).

Both positive and negative edges were included in network analysis. Positive edges were included if two nodes had a Pearson’s R correlation >0.5. Similar functional network modeling experiments have used a range of edge thresholds spanning from *R* = 0.3 (Orsini et al., 2018) to *R* = 0.87 (Wheeler et al., 2013). We chose an edge threshold of *R* = 0.5 to ensure that all nodes within the network had at least one edge with one other node (Qi et al., 2014; Gao et al., 2019; Kimbrough et al., 2020). Negative edges were also included in cases where two nodes had a Pearson’s R correlation <−0.5. To our knowledge, this is the first experiment to measure c-Fos brain activity to included negative edges in network analysis.

To characterize individual nodes within the network, two metrics were used that reflect within-module connectivity and between-module connectivity. The within-module degree z score is a commonly used measure of the level of connectivity between one node and other nodes within the same module (Guimera and Nunes Amaral, 2005). Here, we developed a modified within-module degree variable that better reflects within-module connectivity for small network models. Our modified within-module degree z score (mWMDz) differs from the original in that it is calculated using the strength of the edges between multiple nodes instead of the number of edges between nodes. The mWMDz for node *i*, Eq. 1, is defined as:


(1) $$m\,W\,M\,D\,z=\sum_{e_{i}=1}^{K_{i}}\frac{\left|r_{e_{i}}\right|}{\left(s_{i}-1\right)}$$

*r*_*e_i*_ is the Pearson’s *R* values of edge *e*_*i*_ between node *i* and another node in the same module, *s*_*i*_ is the total number of nodes within module *s*, and *K*_*i*_ is the number of edges between node *i* and all other nodes within the same module. The mWMDz has a range of 0 to 1 with 0 reflecting no within-modular connectivity and 1 reflecting maximum within-modular connectivity. This range is achieved by using the absolute values of *r*_*e_i*_, as our network analysis includes both positive and negative edges. If a node is in a module by itself, that node has a mWMDz of 0 by default *K*_*i*_ is the number of edges between node *i* and all other nodes within the same module.

The participation coefficient is a measure of how distributed the edges of a node are between multiple modules (Guimera and Nunes Amaral, 2005). Participation coefficient values range from 0 to 1 with 0 reflecting no participation and values approaching 1 reflecting greater participation. If a node only has edges with other nodes in the same module, it will have a participation coefficient value of 0. Alternatively, if a node’s edges are spread equally between many modules, that node will have a participation coefficient value close to 1. For participation coefficient, Eq. 2, *K*_*is*_ (between-module degree) is the number of edges (both positive and negative) between node *i* and nodes in module *s. k_*i*_* (total degree) is the number of edges (both positive and negative) between node *i* and all other nodes within the network. The participation coefficient of each node, *PC* is then defined as:


(2) $$P\,C=1-\sum_{s=1}^{N_{M}}{\left(\frac{K_{i\,s}}{k_{i}}\right)}^{2}$$

All variables were calculated in python and then modeled in the Gephi 0.9.2 software package (Bastian et al., 2009).

## Results

### Characterization of Affiliative and Aggressive Behaviors

Male prairie voles that were pair-bonded for 2 weeks exhibited different behavioral responses toward varying social stimuli presented. Male voles spent significantly more time interacting with the female stranger compared to the partner and stranger male (*p* < 0.05, Figure 1C). Further, males were predominantly affiliative when interacting with their partners and mainly aggressive when interacting with strangers (*F*_(2,27)_ = 9.98, *p* < 0.001; Figures 1B–D). The S-N-K test indicated that males exposed to an opposite-sex stranger displayed significantly more olfactory investigation, threat aggression (chases, offensive upright postures, lunges, etc.), and offensive aggression (attacks, bites, retaliatory attacks, etc.) compared to the partner. Male voles also displayed significantly more overall aggression (threat and offensive aggression) when exposed to a same-sex stranger compared to the partner (threat; *F*_(2,27)_ = 4.68, *p* < 0.02; offensive; *F*_(2,27)_ = 8.86, *p* < 0.001; Figures 1F–H). Our results of increased overall aggression toward the stranger conspecifics replicates previous results shown in Winslow et al. (1993) and Gobrogge et al. (2007). There were also group differences found in affiliative behaviors (*F*_(2,27)_ = 12.24, *p* < 0.0002, Figure 1E). Based on the S-N-K test, male voles exhibited significantly higher levels of affiliative behaviors toward their partner as compared to both the female and male stranger.

### Regional Activity Associated With Resident Exposure

**FIGURE 2:**
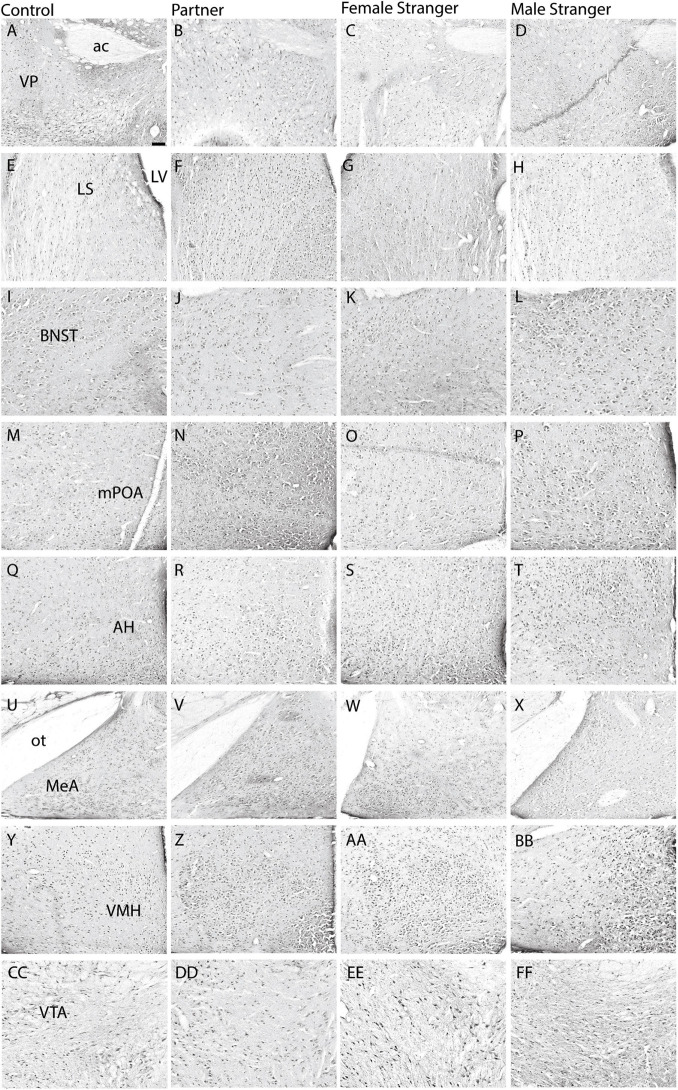
Representative images are displayed for each condition per region in order from control **(A,E,I,M,Q,U,Y,CC)**, partner **(B,F,J,N,R,V,Z,DD)**, opposite-sex stranger **(C,G,K,O, S,W,AA,EE)**, and same-sex stranger **(D,H,L,P,T,X,BB,FF)** (ac. anterior commissure; LV. lateral ventricle; ot. optic tract). Scale bar = 100 μm in control images (applies to all other conditions).

**FIGURE 3:**
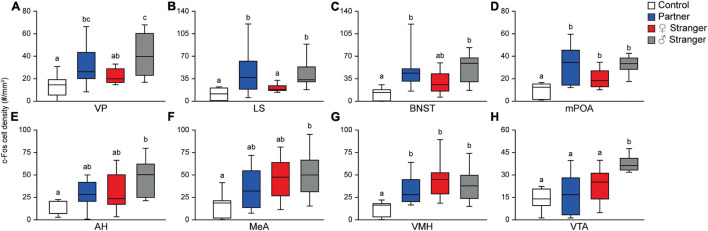
Pair-bonded male prairie voles exposed to social stimuli had an increased density of c-Fos expression in eight of the thirteen regions presented in the SDM network. Compared to male prairie voles not exposed to a social stimulus, there was a higher density of Fos-positive cells for one of the three social stimuli in the ventral pallidum (VP; **A**), lateral septum (LS; **B**), bed nucleus of the stria terminalis (BNST; **C**), medial preoptic area (mPOA; **D**), anterior hypothalamus (AH; **E**), medial amygdala (MeA; **F**), ventromedial hypothalamus (VMH; **G**), and ventral tegmental area (VTA; **H**). Groups labeled with unique alphabetical letters differed significantly by *post hoc* SNK test in which a significant main effect was detected in the ANOVA (*p* < 0.05). If groups share any letter, there was no significant difference between them. Thus, “a” was different from “b,” but “ab” was equal to both “a” and “b” (control, *n* = 7; partner, *n* = 9; opposite-sex stranger, *n* = 9; same sex stranger, *n* = 9).

The immediate early gene, c-Fos is an established marker for neuronal activity and resulted in dense nuclear staining in eight of the thirteen brain regions associated with the SDMN (Chung and Brief, 2015). The resident exposure test induced c-Fos density increases in regional-specific and social stimulus-specific manners. First, group differences in c-Fos density were observed in the mPOA (*F*_(3,30)_ = 8.22, *p* < 0.0004; Figures 2M–P, 3D) and VMH (*F*_(3,30)_ = 4.45, *p* < 0.01; Figures 2Y–BB, 3G). c-Fos density differences were observed in the partner and same sex stranger in the VP (*F*_(3,30)_ = 5.16, *p* < 0.005; Figures 2A–D, 3A), LS (*F*_(3,30)_ = 5.19, *p* < 0.005; Figures 2E–H, 3B), and BNST (*F*_(3,30)_ = 5.64, *p* < 0.003; Figures 2I–L, 3C). Lastly, male voles exposed to same sex stranger induced a c-Fos density increase the AH (*F*_(3,30)_ = 5.52, *p* < 0.004; Figures 2Q–T, 3E), MeA (*F*_(3,30)_ = 3.04, *p* < 0.04; Figures 2U–X, 3F), and VTA (*F*_(3,30)_ = 9.91, *p* < 0.0001; Figures 2CC–FF, 3H). The S-N-K *post hoc* test indicated that male subjects exposed to one of the three social stimuli had increased c-Fos expression compared to baseline. Based on the S-N-K test, the increased c-Fos density is associated with the resident social exposure test. Our results show of c-Fos density increases in the BNST, mPOA, AH, MeA, and VTA shows similarity to previous research such as: Cushing et al. (2003), Curtis and Wang (2003), Gobrogge et al. (2007), and Wang et al. (1997).

### Neural Coactivation Caused by Resident Exposure Test

**FIGURE 4:**
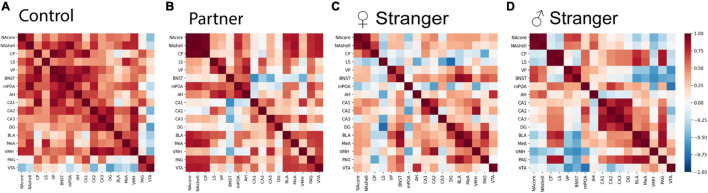
Interregional Pearson correlation heat maps for each social exposure. **(A)** Correlation heat map for control, **(B)** partner exposure, **(C)** opposite-sex stranger, and **(D)** same sex stranger (control, *n* = 7; partner, *n* = 9; opposite-sex stranger, *n* = 9; same sex stranger, *n* = 9).

To examine if various social exposures result in changes in brain activity and organization, we assessed the changes of neural coactivation of the SDMN and modular structuring as compared to the non-social exposed male voles. We examined the interregional coactivation of all regions using c-Fos correlations for each social exposure type. When first examined, there are clear differences between individual social and non-social exposure types (Figure 4). Specifically, the control voles showed higher resting-state coactivation throughout the network, where the voles interacting with varying social stimuli fewer positive correlations across the entire network, but instead, there were unique positive and negative coactivation patterns based on the social stimulus encountered (Figure 4A). When assessing coactivation patterns between social exposure types, the partner (Figure 4B) encounter increased positive coactivation patterns compared to both the opposite-sex and same-sex stranger (Figures 4C,D). Also, we see increased negative coactivation patterns for the same-sex stranger compared to the partner or opposite-sex stranger. Overall, these data suggest that distinct coactivation patterns are displayed in male voles depending on the social exposure and when re-exposed to their partner presents the strongest coactivation pattern between regions in the network.

### Exposures to Varying Stimuli Results in Distinct Modular Structuring in the Social Decision-Making Network

**FIGURE 5:**
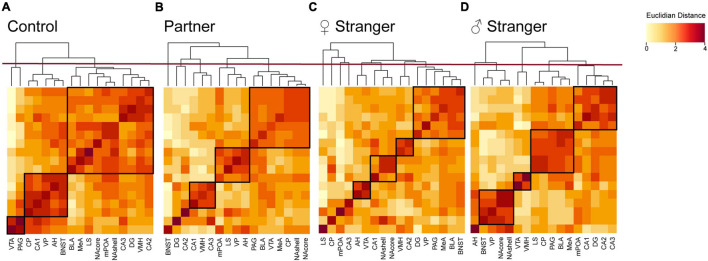
Hierarchical clustering for each social exposure. Modules were determined by cutting each dendrogram at half of the maximal tree height (displayed by red line). Modules for each exposure are boxed in black. **(A)** Control dendrogram displayed three distinct modules of coactivation. **(B)** Partner exposure dendrogram displayed five distinct modules of coactivation. **(C)** Opposite-sex stranger dendrogram displayed eight distinct modules of coactivation. **(D)** Same sex stranger dendrogram displayed five distinct modules of coactivation.

Pearson’s correlation matrix allows for the observation of interregional coactivation as previously mentioned. However, this analysis only allows for the observation between two individuals regions. To examine the coactivation of a cluster of regions or regions that form modules, we used hierarchical clustering. Hierarchical clustering allowed for the observation and identification of the modular organization of the SDMN in non-social exposed control voles as well as the male voles exposed to one of three social exposures. Overall, the control voles displayed the least number of modules, indicating a general integration of connectivity in the SDMN during resting-state. By comparison, all social encounters remodeled the regional clustering and led to more restricted modules, with the exposure to the opposite-sex stranger generated the greatest number of modules including a number of regional isolates. We also found that there were no conserved modules across the social stimuli. Specifically, in control voles the network was organized into 3 distinct modules (Figure 5A). In the male voles re-exposed to their partner, we found that the network was organized into 5 distinct modules with one of the modules being an isolate (Figure 5B). Male voles exposed to an opposite-sex stranger demonstrated an increase, with a total of 8 modules and four of those modules are isolates (Figure 5C). Lastly, male voles exposed to a same-sex stranger displayed 5 total modules with a single isolate presented (Figure 5D). These data indicate that male voles present distinct modular structuring of the SDMN depending on the social exposure.

### Network Analysis With the Use of Graph Theory

To further characterize our networks compared to the hypothesized SDMN, we used a graph theory approach. Refer to the methods section for specific calculations and terms. In short, we examined positive and negative connections, based on the Pearson’s correlation coefficient (threshold > ± 0.5R), between regions and modules displayed from the hierarchical clustering. We also determined the participation coefficient (strength of intermodular connectivity) and within-module degree Z-score (strength of intramodular connectivity) for all regions within the network.

**FIGURE 6:**
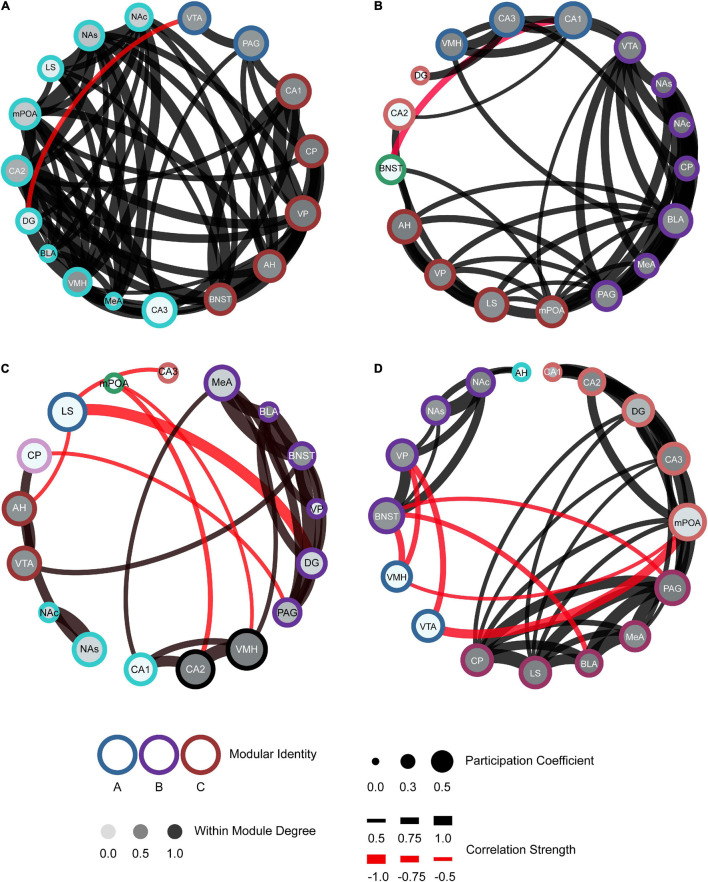
Functional connectivity mapping for each social exposure: control **(A)**, partner **(B)**, opposite-sex stranger **(C)**, and same-sex stranger **(D)**. Nodes/brain regions for each network are represented by circles. The outer color of each circle represents modular identity. The shade of the internal color for each circle represents the within-module degree score (white = lowest score; black = highest score). The size of each circle represents the participation coefficient (smaller = Lower participation coefficient, larger = higher participation coefficient). The thickness of each line between nodes represents the strength of the correlation between regions (thin = lower correlation; thick = higher correlation). Positive (black) and negative (red) lines are represented between nodes based off the correlation matrices.

The generation of these network models allows for better visualization of the functional connectivity of the SDMN during distinct social encounters beyond the correlational matrices and dendrograms (Figure 6; mWMDz and PC values are available in extended data Figure 6). Our network models suggest that male voles re-exposed to their partner had an increase in connectivity and organization as compared to the stranger conspecifics (Figure 6B). The partner network also demonstrates an overall increase in the participation coefficient and within-module degree Z-score as compared to the stranger conspecific networks. The opposite-sex stranger displays decreased connectivity and organization compared to the partner and same-sex stranger (Figure 6C). Both stranger networks demonstrated greater negative connections between modules compared to the resting-state or partner-evoked networks. This is most prominent in the same-sex stranger network (Figure 6D). Together, these data indicate that there are notable connective and modular differences within the functional connectivity networks based on the social stimulus presented.

## Discussion

Social living presents a variety of distinct social interactions which elicit neural systems for recognition, motivation, attraction, and other cognitive processes to enact adaptive behavior (Smith et al., 2013). The present study used a long-term male-female cohabitation paradigm in prairie voles to assess context-appropriate behaviors and the functional connectivity of the SDMN during various social encounters. Pair-bonded males displayed selective affiliation toward their partner and selective aggression toward both opposite-sex and same-sex stranger conspecifics, consistent with published literature (Winslow et al., 1993; Gobrogge et al., 2007). These data indicate that pair-bonded male voles can differentiate between their mate and a stranger conspecific as well as display context-appropriate social behaviors. Furthermore, social encounters evoked increased activity in eight of the thirteen regions associated with the SDMN in pair-bonded male voles when exposed to one of three social stimuli. Specifically, while the VMH increased activity after all social encounters, the mPOA, BNST, LS, and VP displayed increased c-Fos levels only when exposed to their partner or same-sex stranger. Each of these regions have been implicated in regulating both affiliation and aggression (Liu et al., 2001; Lim and Young, 2004; Lei et al., 2010; Vogel et al., 2018). In addition, the AH, MeA, and VTA had increased c-Fos expression when exposed to the same-sex stranger, similar to previous literature (Gobrogge et al., 2007). Both the AH and VTA have been implicated in regulating selective aggression, along with the MeA regulating approach-avoidance behaviors (Curtis and Wang, 2005; Gobrogge et al., 2017; Tickerhoof et al., 2020). This evoked regional activity may demonstrate the neural response toward conspecifics that is necessary to process relevant social information and generate adaptive behaviors.

Of course, the SDMN hypothesis predicts that behavior is the product of interregional coactivity across a network of regions rather than activity of any individual brain region. Several network modeling studies have associated neural network connectivity to behavior, including aggression (Teles et al., 2015), positive social interactions (Rilling et al., 2018), and mating (Johnson et al., 2016; Kabelik et al., 2018). In this study, patterns of coactivation across regions were measured using correlational models, hierarchical cluster analysis, and graph theory-derived network modeling. Our networks demonstrated particularly interesting node-to-node and modular connections among brain regions that have been implicated in regulating social behavior of prairie vole and other species. Specifically, in the partner network, one module that included the AH, LS, mPOA, and VP incorporated many of the regions suggested to be in the social behavioral network (Newman, 1999; O’Connell and Hofmann, 2011). These four regions have all been implicated in certain affiliative behaviors in prairie voles (Liu et al., 2001; Curtis and Wang, 2003; Lim and Young, 2004; Gobrogge et al., 2007, 2009). In addition, one study conducted in male prairie voles looked at c-Fos levels in a subset of SDMN regions incorporated into an alternative network called the “pair bonding network” during sociosexual interactions (Johnson et al., 2016). This network included the NAs, NAc, MeA, BLA, and mPOA, and all of these regions were correlated during social interactions. Our partner network features correlations across all of these regions as well. Furthermore, a recent fMRI study in voles reported that functional connectivity of a neural network correlates with partner-directed affiliation (López-Gutiérrez et al., 2021). Our results show similar regional coactivity as well as modular organization suggesting that such interconnected regions may be correlated with affiliative behaviors (e.g., huddling) and reward-processing (e.g., mating).

In the opposite-sex stranger network, there was a negative connection between the AH to LS, compared to a positive correlation between these regions in the partner network. The AH and LS have been shown to have bidirectional axonal projections that play a role in affiliation behaviors (Staiger and Wouterlood, 1990). The AH was also associated with the VTA in the stranger network. This is interesting as the AH and VTA play roles in vole aggression (Greenwell et al., 2002; Curtis and Wang, 2005). This may suggest that the activity between these regions is coordinated and gives rise to stranger-directed aggression. Furthermore, the mPOA, AH, and VMH are part of the “hypothalamic attack areas” (Roeling et al., 1994). Each of these regions were active during same-sex stranger encounters, and the mPOA and VMH were connected in the network. While AH was not directly connected to these two regions, it was connected to limbic regions (BNST and NAcc) that promote positive valence to aggression. In addition, there were functional connections from the BNST, MeA, and VMH, and interactions across these regions are suggested to regulate approach-avoidance of threatening stimuli (Miller et al., 2019). The identification of interconnected circuits in our networks provides insight into how these brain regions work as a network during these social encounters to potentially either promote or suppress affiliative or aggressive behaviors.

One of the goals of this work was to generate functional connectivity neural networks that are formed during social interactions and compare them to the theorized SDMN model in order to determine its validity. The SDMN consists of two interconnected circuits including: the social behavioral network and mesolimbic reward system (O’Connell and Hofmann, 2011). Interestingly, our partner network displays strong overlap with the theorized SDMN model. Within the partner network, the module that includes the AH, LS, mPOA, and VP incorporates three of the seven regions suggested to be in the social behavioral network. As for the BNST, PAG, and MeA, the previously mentioned module has direct connections to associated modules, the region, or both. The VMH is the only region of the social behavior network that does not have a direct projection to the aforementioned module but does have direct projections to the module with the MeA and PAG. As for the mesolimbic reward system, one module contains four associated regions (BLA, CP, NAcc, and VTA) and direct connections to the remaining regions besides the BNST and two nuclei of the HIP [CA2 and dentate gyrus (DG)]. However, both stranger networks failed to display modules that could be classified as the hypothesized social behavioral network or mesolimbic reward system. Rather the functional connectivity of the stranger-derived networks was unique in organization and included a number of negative correlations. Negative or opposite coactivation patterns are common in the SDMN during agonistic encounters as this has been reported in other species, including zebrafish (Teles et al., 2015) and brown anoles (Kabelik et al., 2018). In these studies, agonistic behaviors were also negatively associated to SDMN interregional connectivity. Thus, there may be specific regions antagonizing or suppressing other regional activity, and network function as a whole, during agonistic encounters.

Understanding the neural mechanism by which animals detect and respond to socioemotional cues is an important area of neurobiology. Prairie voles and other social species navigate their environment engaging in various social interactions that impact territorial boundaries, resource allocation, social status, interpersonal dynamics, and social attachments (Carter et al., 1995). Thus, it is detrimental to their social lives to be adaptive and responsive to these distinct social encounters and display context-appropriate social behaviors (Chen and Hong, 2018). Functional connectivity, defined as correlated activation of spatially distinct neural activity, is one of the first steps in understanding the theoretical SDMN (Park and Friston, 2013). This type of analysis is widely used in fMRI work. However, by adapting the mathematical principles from these studies, we can use this analysis in our study of freely moving animals and IEGs. Our regional coactivation data showed high, global connectivity at resting-state, similar to a recent functional magnetic resonance imaging (fMRI) study (Ortiz et al., 2018) and another c-Fos mapping study (Johnson et al., 2016) in prairie voles. This may be, in part, due to low c-Fos expression in many regions when environmental stimulus is absent. The true benefit from the c-Fos mapping approach compared to fMRI is that we were able to observe the remapping of interregional connectivity across the SDMN following social encounters which revealed distinct regional composition and modular organization based on type of social encounter. This has brought insight into the organization of this network and potentially what underlies the context-appropriate behaviors during distinct social encounters that maintain social attachment and commitment in prairie voles.

Collectively, this work begins to reveal the organization of the “social brain” that underlies the context-appropriate behaviors during distinct social encounters that maintain social attachment and commitment in prairie voles. There are limitations to functional connectivity modeling such as the inability to correlate neural activation to specific behavioral output and understanding specific neuromodulator promoting these behavior outputs. Importantly, global or site-specific pharmacology or other methods of manipulation combined with this network modeling approach will further our understanding of SDMN dynamics and behavioral output. For the SDMN to be a genuine neural network, it must display structural connectivity, functional specialization, and functional connectivity. Future research should also incorporate structural mapping of the SDMN, along with determining how the network organization regulates behavioral outputs during various social exposures.

## Data Availability Statement

The raw data supporting the conclusions of this article will be made available by the authors, without undue reservation.

## Ethics Statement

The animal study was reviewed and approved by the Institutional Animal Care and Use Committee at the University of Kansas.

## Author Contributions

KG, BG, APS, and AS contributed to conception and design of the study. KG and BD organized the database. KG, BD, AK, and AS performed the statistical analysis. KG wrote the first draft of the manuscript. BD and AS wrote sections of the manuscript. All authors contributed to manuscript revision, read, and approved the submitted version.

## Conflict of Interest

The authors declare that the research was conducted in the absence of any commercial or financial relationships that could be construed as a potential conflict of interest.

## Publisher’s Note

All claims expressed in this article are solely those of the authors and do not necessarily represent those of their affiliated organizations, or those of the publisher, the editors and the reviewers. Any product that may be evaluated in this article, or claim that may be made by its manufacturer, is not guaranteed or endorsed by the publisher.

## Footnotes

1 Commitment signaling refers to the verbal and non-verbal gestures an individual will express to protect and strengthen a bond. These gestures include words of affirmation, gestures of affection and courtship, and one’s choice to ignore or reject other potential partners (Gonzaga et al., 2001).
