# Supplementary material for: Pair Bond-Induced Affiliation and Aggression in Male Prairie Voles Elicit Distinct Functional Connectivity in the Social Decision-Making Network
Source: Front Neurosci. 2021 Oct 15;15:748431. doi: 10.3389/fnins.2021.748431 (PMC8553992; doi:10.3389/fnins.2021.748431)
Supplement: Supplementary file 1 [file Table_1.docx]

| **Brain Region** | **Abbreviation** | **Control PC** | **Partner PC** | **Unfamiliar Female PC** | **Unfamiliar Male PC** |
| --- | --- | --- | --- | --- | --- |
| Nucleus accumbens core | NAcore | 0.35 | 0.24 | 0.00 | 0.38 |
| Nucleus accumbens shell | NAshell | 0.42 | 0.24 | 0.50 | 0.38 |
| Caudate putamen | CP | 0.50 | 0.24 | 0.50 | 0.49 |
| Lateral septum | LS | 0.28 | 0.61 | 0.67 | 0.49 |
| Ventral pallidum | VP | 0.58 | 0.48 | 0.00 | 0.48 |
| Bed nucleus of the stria terminalis | BNST | 0.48 | 0.44 | 0.28 | 0.61 |
| Medial preoptic area | mPOA | 0.46 | 0.51 | 0.00 | 0.67 |
| Anterior Hypothalamus | AH | 0.49 | 0.61 | 0.50 | 0.00 |
| Hippocampus subnucleus CA1 | CA1 | 0.57 | 0.72 | 0.44 | 0.00 |
| Hippocampus subnucleus CA2 | CA2 | 0.44 | 0.50 | 0.67 | 0.32 |
| Hippocampus subnucleus CA3 | CA3 | 0.57 | 0.63 | 0.00 | 0.50 |
| Hippocampus subnucleus dentate | DG | 0.28 | 0.00 | 0.38 | 0.49 |
| Basolateral amygdala | BLA | 0.00 | 0.61 | 0.00 | 0.32 |
| Medial amygdala | MeA | 0.00 | 0.24 | 0.56 | 0.44 |
| Ventromedial hypothalamus | VMH | 0.38 | 0.50 | 0.75 | 0.44 |
| Periaqueductal grey | PAG | 0.56 | 0.48 | 0.32 | 0.59 |
| Ventral tegmental area | VTA | 0.50 | 0.49 | 0.50 | 0.50 |

**Supplemental Table 1.** Participation coefficient (PC) values for each network model.

| **Brain Region** | **Abbreviation** | **Control mWMD_Z_** | **Partner mWMD_Z_** | **Unfamiliar Female mWMD_Z_** | **Unfamiliar Male mWMD_Z_** |
| --- | --- | --- | --- | --- | --- |
| Nucleus accumbens core | NAcore | 0.39 | 0.86 | 0.23 | 0.78 |
| Nucleus accumbens shell | NAshell | 0.47 | 0.85 | 0.23 | 0.67 |
| Caudate putamen | CP | 0.89 | 0.82 | 0.00 | 0.78 |
| Lateral septum | LS | 0.21 | 0.69 | 0.00 | 0.81 |
| Ventral pallidum | VP | 0.91 | 0.73 | 0.11 | 0.68 |
| Bed nucleus of the stria terminalis | BNST | 0.87 | 0.00 | 0.73 | 0.68 |
| Medial preoptic area | mPOA | 0.43 | 0.66 | 0.00 | 0.16 |
| Anterior Hypothalamus | AH | 0.80 | 0.80 | 0.74 | 0.00 |
| Hippocampus subnucleus CA1 | CA1 | 0.82 | 0.68 | 0.00 | 0.47 |
| Hippocampus subnucleus CA2 | CA2 | 0.60 | 0.00 | 0.87 | 0.78 |
| Hippocampus subnucleus CA3 | CA3 | 0.15 | 0.71 | 0.00 | 0.80 |
| Hippocampus subnucleus dentate | DG | 0.23 | 0.00 | 0.23 | 0.45 |
| Basolateral amygdala | BLA | 0.36 | 0.71 | 0.79 | 0.83 |
| Medial amygdala | MeA | 0.41 | 0.75 | 0.29 | 0.75 |
| Ventromedial hypothalamus | VMH | 0.77 | 0.75 | 0.87 | 0.00 |
| Periaqueductal grey | PAG | 0.74 | 0.73 | 0.46 | 0.84 |
| Ventral tegmental area | VTA | 0.74 | 0.76 | 0.74 | 0.00 |

**Supplemental Table 2.** Modified within-module degree z-score (mWMD_Z_) values for each network model.
